# Invasive cribriform carcinoma of the breast presenting as an erythematous papule on the nipple: A case report

**DOI:** 10.1002/ccr3.9055

**Published:** 2024-06-04

**Authors:** Po‐Yu Chen, Yu‐Hsuan Ho, Chih‐Jung Chen, Chien‐Shan Chiu

**Affiliations:** ^1^ Department of Medical Education Taichung Veterans General Hospital Taichung Taiwan; ^2^ Department of Dermatology Taichung Veterans General Hospital Taichung Taiwan; ^3^ Department of Pathology and Laboratory Medicine Taichung Veterans General Hospital Taichung Taiwan

**Keywords:** breast, invasive cribriform carcinoma, mammary Paget's disease, nipple, papule

## Abstract

Invasive cribriform carcinoma (ICC) is a rare form of invasive breast carcinoma with good prognosis. To date, case reports considering skin manifestations of ICC are scarce. We herein report a case of pure ICC presenting as an erythematous papule on the nipple with mammary Paget's disease in the epidermis. We aim to bring awareness to skin manifestation of ICC.

## 1 INTRODUCTION

Invasive cribriform carcinoma (ICC) of the breast, initially described by Page et al. in 1983 due to its predominant cribriform growth pattern in the invasive component and relatively favorable prognosis, is a rare type of breast carcinoma, accounting for 0.3% to 3.5% of primary breast carcinomas.^1^, ^2^ ICC patients typically have a small tumor size, few axillary lymph node invasion, low distant metastasis rate, tumor with a high frequency of well differentiation, high ER and PR positive rates with rarely‐observed human epidermal growth factor receptor 2 (HER2) amplification, and a low proliferation index.^3^, ^4^, ^5^ To date, studies of skin manifestations of invasive cribriform carcinoma of the breast are notably scarce. We herein report a patient of ICC with a presentation as an erythematous papule on the nipple that no one has reported before.

## 2 CASE HISTORY/EXAMINATION

**FIGURE 1:**
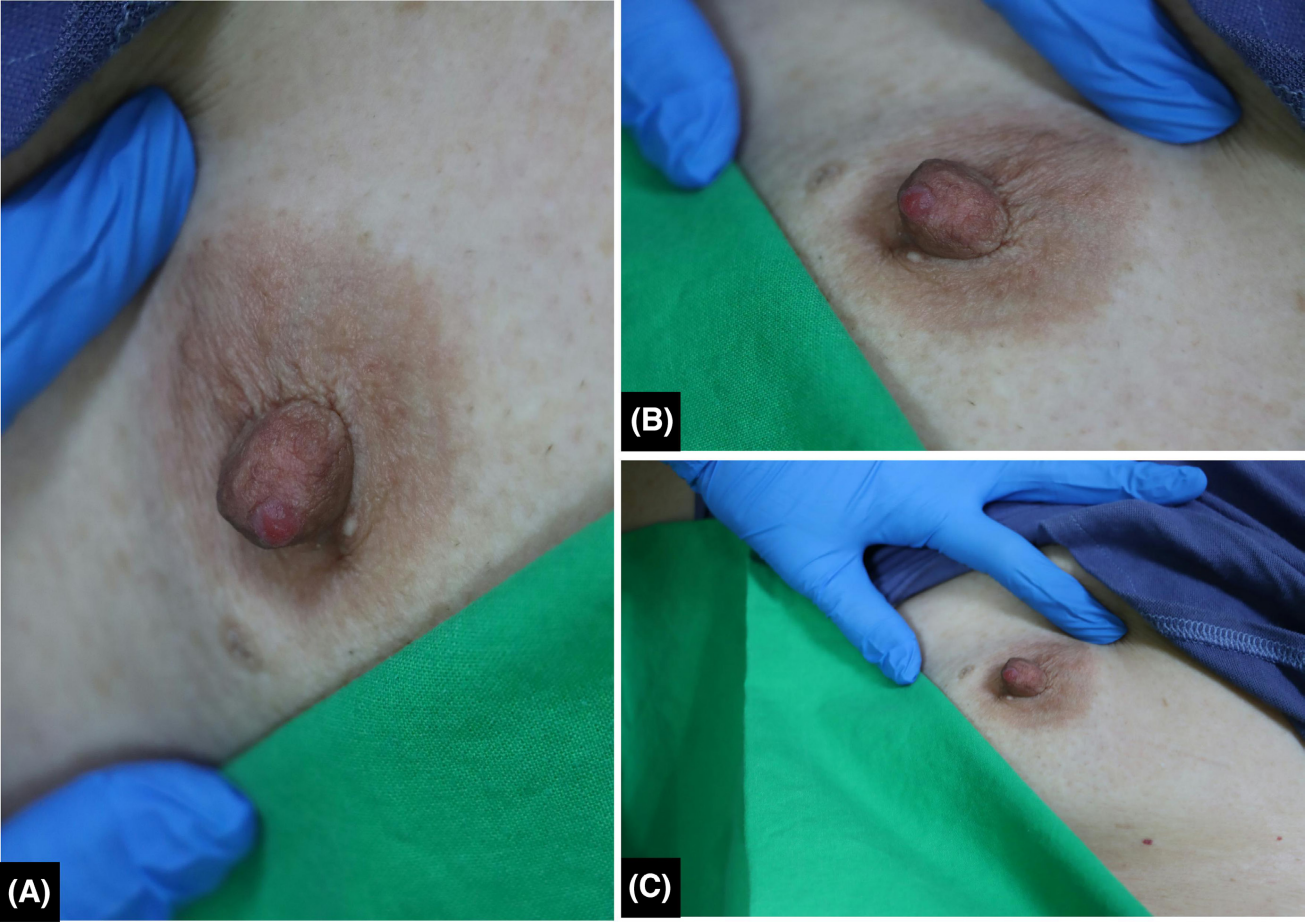
(A–C) Clinical photograph showing a well‐demarcated, centrally erythematous with violaceous in the periphery and dome‐shaped papule on the patient's right nipple tip.

A 71‐year‐old Asian woman visited our Department of Dermatology with a chief complaint of a reddish protruding skin lesion on the tip of her right nipple that had appeared for 6 months. Upon physical examination, the skin lesion was a solitary, well‐demarcated papule, 0.2 × 0.2 cm in size, appearing centrally erythematous with violaceous in the periphery, fairly dome‐shaped and firm (Figure 1A–C). Besides the presence of the papule, the patient did not report any symptoms at the nipple or any other abnormal symptoms. Her medical history was also unremarkable.

## 3 DIFFERENTIAL DIAGNOSIS AND INVESTIGATIONS

**FIGURE 2:**
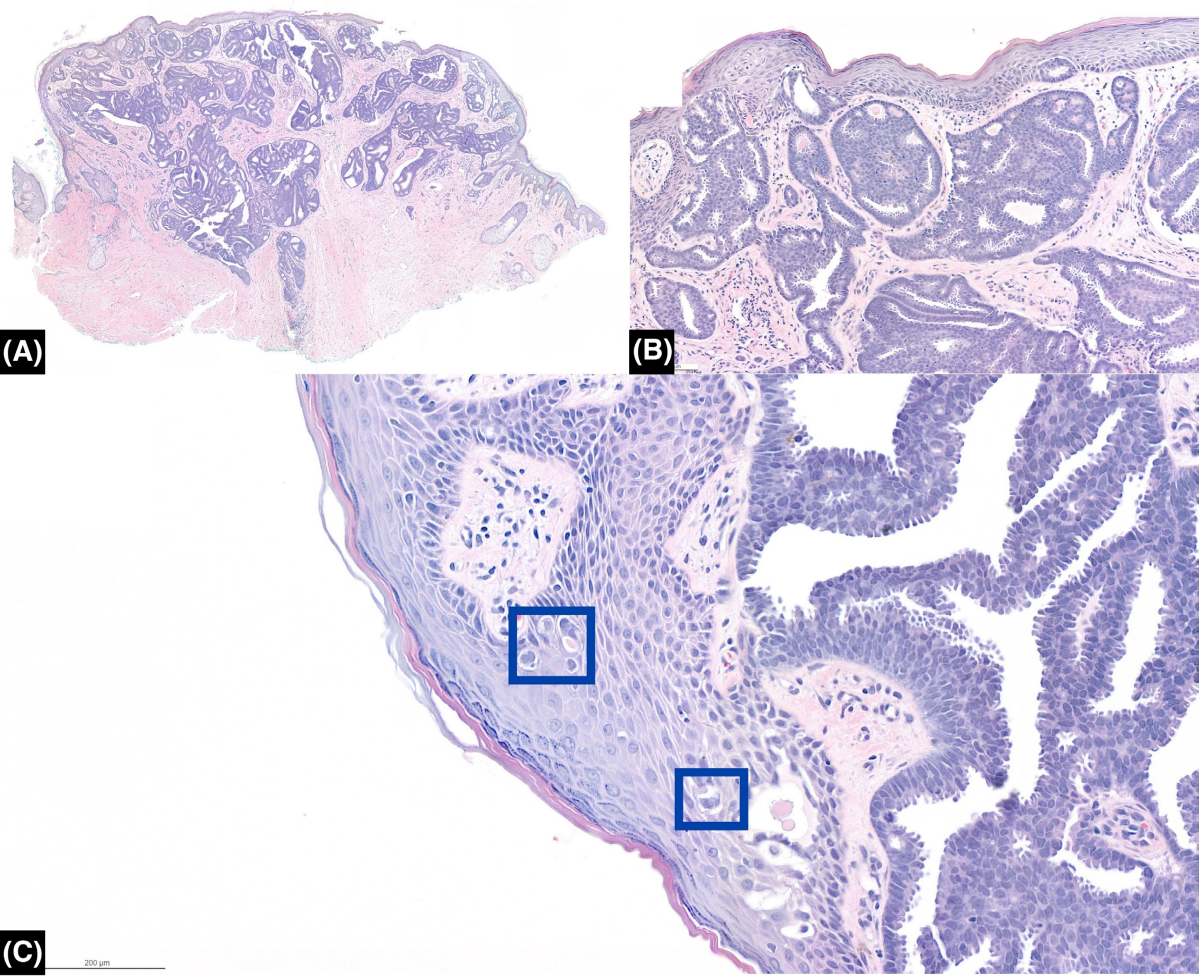
(A) Pathological results revealed invasive cribriform carcinoma of the breast in H&E staining. (B) More than 90% of tumor cells were arranged in nest‐like groups with a cribriform growth pattern in pure invasive cribriform carcinoma (ICC). (C) Single and small cluster of Paget cells (box) with vesicular nucleus and prominent nucleolus in the epidermis indicated mammary Paget's disease (MPD).

**FIGURE 3:**
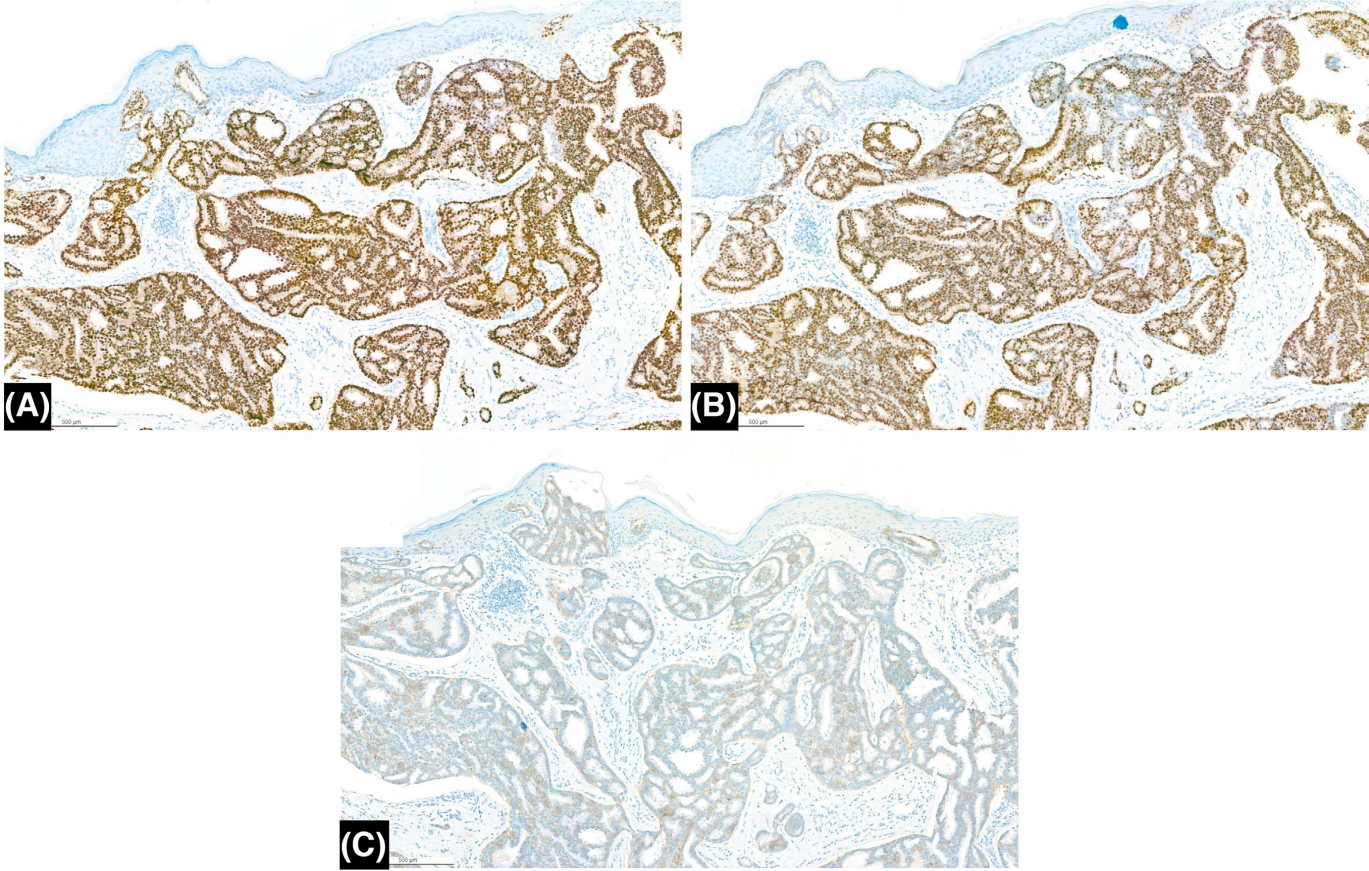
(A) ER showed strong nuclear staining. (B) PgR showed strong nuclear staining. (C) Human epidermal growth factor receptor 2 (HER2) protein expression scored as 0.

With these clues, a benign skin lesion had been impressed. Therefore, a local excision was performed to obtain a specimen of 0.7 × 0.5 × 0.3 cm in size. Biopsy results turned out to be pure invasive cribriform carcinoma of the breast (pure ICC), characterized by the invasive cribriform pattern comprising >90% of the lesion, in addition to mammary Paget's disease (MPD) in the epidermis (Figure 2A–C). Immunohistochemical tests showed positive expressions of both estrogen and progesterone receptors (ER >95%, PgR >95%). The immunohistochemical reaction to human epidermal growth factor receptor type 2 (HER2) protein was negative (HER 2 [−]) (Figure 3A–C). The Ki‐67 labelling index was 5%.

## 4 TREATMENT, OUTCOME, AND FOLLOW‐UP

In the diagnosis of pure ICC with MPD, she was referred to the Breast Surgery Department for further survey and treatment. Results on both CEA and CA15‐3 levels were normal. Bilateral breast mammography (CC and MLO view) revealed segmental fine pleomorphic microcalcifications in the lower‐inner quadrant of her right breast. Breast ultrasound revealed two oval hypoechoic lesions with tiny calcifications which were 7.8 and 24.5 mm in size at the right breast near the areola. These findings raised high suspicion for malignancy and were consistent with a classification of 4C in the BIRADS‐US system. Whole body bone scan found no evidence of bone metastasis. With a previous biopsy pathology report revealing ICC and MPD, a full discussion was carried out with the patient. Finally, a simple mastectomy and sentinel lymph node biopsy were performed on her right breast.

With the right side simple mastectomy, histopathology of the surgical specimen showed multifocal intermediate‐grade ductal carcinoma in situ (DCIS) measuring up to 9 mm located at 4 o'clock position and 1 cm from the right nipple‐areolar complex (NAC), and an intraductal papilloma without atypia measuring 16 mm located at 6 o'clock position and 2 cm from the right NAC. There was no residual invasive carcinoma seen in the mastectomy specimen. Both frozen sections of sentinel lymph nodes revealed no evidence of metastasis. The pathological TNM stage was pT1aN0 (Stage IA), considering the size of ICC in the excisional biopsy according to the AJCC 8th edition. Further treatment plans for this patient included a 5‐year tamoxifen therapy and regular follow‐up at the Breast Surgery Department.

## 5 DISCUSSION

Based on the clinical features of the papule on her nipple, our initial impressions were benign skin lesions such as pyogenic granuloma, nipple adenoma, Spitz nevus, and intradermal nevus. Nevertheless, these possibilities were easily ruled out due to the cribriform pattern in the excisional biopsy pathology. Importantly, primary cutaneous cribriform apocrine carcinoma (PCCAC), which usually presents as an asymptomatic nodule on the extremities clinically and appearing as a non‐encapsulated, well‐demarcated dermal nodule without epidermal attachment at scanning magnification, should be included in the histopathologic differential diagnosis for it is also composed of a predominantly cribriform component pathologically.^6^ However, the cribriform growth pattern in our biopsy pathology was clearly infiltrating and with epidermal invasion. Furthermore, we found no evidence of apocrine differentiation. The immunohistochemical stains revealed ER >95%, PgR >95%, and negative CK5/6 as well as p63 in the invasive part of the tumor. These features were not compatible with PCCAC. As a result, PCCAC was considered less likely, and the final pathologic diagnosis of ICC was made. Although ICC usually presents as an asymptomatic mass or may even be clinically occult,^1^, ^3^ the chance of ICC presenting as skin lesions cannot be ignored. The case reported by Katarzyna et al., in which ICC mimicked a breast abscess,^7^ along with our case of ICC presenting as a nipple papule, collectively demonstrates ICC's potential to primarily manifest through skin lesions. To the best of our knowledge, ICC is a tumor having a more favorable prognosis than invasive breast carcinoma with less cribriform patterns and low‐grade invasive ductal carcinoma without cribriform structures.^3^ In a study by Thennavan et al.,^8^ TCGA‐BRCA was classified into 12 consensus groups based on integrated genomic and histological features. These 12 consensus groups were further organized into four broader groups using their previously published breast epithelial differentiation score (D score) to facilitate genomic analyses. ICC was categorized as the high differentiation group, which presented special histologies with high D scores. In this group, a paucity of 22q loss, 20q gain, TP53 mutation, and an increased GATA3 mutation were identified as uniting features. In addition, a trend of decrease in copy number alterations events was observed as the degree of differentiation of a histologic type increased. Most of these genomic findings support the favorable biology of ICC, consistent with several previous studies regarding genetics aberrations and the prognosis of breast cancer. Despite favorable prognosis of ICC, there are still chances of aggressive behavior or development into an advanced stage if left untreated long enough. Mishra et al. reported a case of ICC with extensive perineural infiltration and lymphovascular invasion in a 58‐year‐old female; and Zhang et al. also reported a case of pure ICC with bone metastasis when left untreated for 13 years.^9^, ^10^ Therefore, such manifestation of a papule on the nipple, as in our case, may result in delayed diagnosis and treatment to ICC. It is crucial for dermatologists to have early awareness about skin manifestation of ICC and to avoid misdiagnosis.

MPD, first described by Sir Paget in 1874 as eczematous nipple and areola skin lesions, is a rare form of breast carcinoma comprising 1% to 3% of all breast cancers. Nearly all cases of MPD are affiliated with underlying breast carcinomas, typically located near the areola, and >90% are either DCIS or invasive ductal carcinomas (IDCs). Pathophysiologically, there are two main hypotheses proposed for the origin of MPD: the epidermotropic theory and the malignant transformation theory. The epidermotropic theory states that Paget cells originate from underlying breast carcinoma, and they migrate via the lactiferous ducts to the epidermis of the nipple. On the other hand, the malignant transformation theory states that Paget cells are malignant transformation of pluripotent keratinocyte stem cells or cells of apocrine gland ducts.^11^ Although remains controversial, it is currently widely accepted that MPD is associated with underlying breast carcinoma, forming the concept based on the epidermotropic theory. In our case, ICC and MPD were diagnosed within the papular lesion on the nipple. This case stands out from >90% of cases in which MPD was diagnosed exclusively with underlying DCIS or IDC. Furthermore, to our best knowledge, only one publication focusing on radiologic characteristics of 52 MPD cases mentioned a case of invasive lobular and invasive cribriform carcinoma being diagnosed with MPD presenting as a mass on mammography,^12^ but details or images were not provided. As to the treatment for MPD plus ICC (MPD‐ICC), current standard treatments often mirror that of “Breast invasive and NAC Paget,” involving mastectomy with axillary staging or breast‐conserving surgery (BCS) followed by whole breast radiation if a clear margin can be achieved.^13^ Given the indolent nature of ICC, some may consider BCS alone without postoperative radiotherapy for MPD plus early‐stage ICC. However, back in the late 20th century, Dixon et al. and Polgar et al. demonstrated local recurrence rates of 33% to 40% following BCS alone in patients with MPD plus DCIS (MPD‐DCIS) and MPD plus IDC (MPD‐IDC).^14^, ^15^ Yao et al. reported in their retrospective study that radiotherapy was associated with significant benefits on 10‐year overall survival for patients with MPD‐DCIS and MPD‐IDC receiving BCS.^16^ In Lin et al.'s meta‐analysis regarding treatment of MPD,^17^ they concluded that except for MPD alone, BCS alone was not recommended for treating MPD‐DCIS and MPD‐IDC given the significant increase in the local recurrence rate when compared to BCS in combination with radiotherapy and mastectomy. Currently, treatment of ICC is based on evidence from IDC. Besides, ICC is certainly more malignant than DCIS. As a result, BCS alone without radiotherapy is not recommended for MPD‐ICC at this point. In our patient, in order to avoid radiation and for fear of the possibility of the lesions on the breast images being multicentric breast cancers, mastectomy with SLNB was performed after sufficient discussion. Additionally, it is worth knowing that the patient was diagnosed with multifocal DCIS at 1 cm near the right NAC in the surgical specimen from simple mastectomy. In our opinion, it was the invasive cribriform carcinoma of the breast but not DCIS resulting in the MPD in the epidermis of the papule (Figure 2C) as consistent with the epidermotropic theory. Although DCIS has been diagnosed near the areola, we believe that the chance of MPD originating from the DCIS was slim. That is because malignant Paget cells were not seen at other parts of the nipple nor at the areola, but merely in the epidermis of the papule on the nipple. Furthermore, based on pathologic images, the gross pictures, and the clinical manifestations of ICC and MPD, we believe that the invasive cribriform carcinoma of breast had caused the dome‐shaped violaceous papule on the nipple, and MPD contributed to the central erythematous part of the papular lesion (Figure 1A). Undoubtedly, more studies are needed to bring attention to the skin manifestation of invasive cribriform carcinoma of the breast. As far as we know, no publication has ever reported on ICC with skin manifestation as a papule on the nipple. Also, no case has been reported with detailed images and descriptions regarding a pure ICC being diagnosed with MPD. In this case report, we aim to bring awareness to skin manifestation of ICC.

## AUTHOR CONTRIBUTIONS

**Po‐Yu Chen:** Conceptualization; investigation; methodology; validation; visualization; writing – original draft; writing – review and editing. **Yu‐Hsuan Ho:** Validation; visualization; writing – original draft; writing – review and editing. **Chih‐Jung Chen:** Resources; supervision; writing – review and editing. **Chien‐Shan Chiu:** Project administration; resources; supervision; writing – review and editing.

## FUNDING INFORMATION

There was no funding for this case report.

## CONFLICT OF INTEREST STATEMENT

There was no potential conflict of interest.

## CONSENT

Written informed consent was obtained from the patient to publish this report in accordance with the journal's patient consent policy.

## Data Availability

Data sharing was not applicable to this article as no datasets were generated or analyzed during the current study.
